# Supplementary material for: Late Cenozoic exhumation in the eastern Junggar Basin: Evidence from zircon (U-Th)/He ages of combustion metamorphic rocks
Source: PLoS One. 2026 May 5;21(5):e0348116. doi: 10.1371/journal.pone.0348116 (PMC13143060; doi:10.1371/journal.pone.0348116)
Supplement: Attachment — Submitted filename: Response to Reviewers.docx [file pone.0348116.s003.docx]

# Response to academic editor

Dear Academic Editor,

We thank you for the editorial evaluation and for the opportunity to revise our manuscript. We have carefully addressed all additional journal requirements requested by PLOS ONE. Our point-by-point responses are provided below.

**Requirement 1**

1 Please ensure that your manuscript meets PLOS ONE's style requirements, including those for file naming.

**Response:**

We have revised the manuscript to comply with PLOS ONE formatting and style requirements (including section structure, headings, and overall presentation). We will also follow PLOS ONE’s file naming requirements upon resubmission and upload the revision files as instructed (Response to Reviewers; Revised Manuscript with Track Changes; Clean Manuscript).

**Requirement 2**

In your Methods section, please provide additional information regarding the permits you obtained for the work. Please ensure you have included the full name of the authority that approved the field site access and, if no permits were required, a brief statement explaining why.

**Response:**

We have added a dedicated subsection in the Methods titled “Fieldwork permits”. In this subsection we clarify that no specific permits were required for field access and sampling, because the work was conducted at accessible outcrops/abandoned or open areas without involving protected sites, protected species, or restricted localities, and all activities complied with local regulations.

(Section 3.1 “Fieldwork permits.”)

**Requirement 3**

Please state what role the funders took in the study. If the funders had no role, please state: "The funders had no role in study design, data collection and analysis, decision to publish, or preparation of the manuscript."

If this statement is not correct you must amend it as needed.

Please include this amended Role of Funder statement in your cover letter; we will change the online submission form on your behalf.

**Response:**

We confirm that the funding agencies had no role in the study design, data collection and analysis, decision to publish, or preparation of the manuscript.

**Requirement 4**

Data Availability Statement: please confirm whether submission contains all raw data required to replicate results (“minimal dataset”). If not, upload as Supporting Information or deposit to a repository.

**Response:**

We confirm that the submission will include the minimal dataset required to replicate all findings in the study. The key underlying numerical data used in our analyses and interpretations (including coal analytical results and single-grain zircon (U–Th)/He data) are provided in the manuscript tables. In addition, to fully comply with PLOS ONE’s minimal dataset policy, we have deposited the **Supporting Information file Table S1**. Statistical results of coal petrographic identification of the B-coal seams in the Xishanyao Formation in the Figshare repository.

**Requirement 5**

Please remove any funding-related text from Acknowledgments and tell us how you would like to update your Funding Statement.

**Response:**

We have removed all funding-related statements from the Acknowledgments section in the revised manuscript. Funding information will be provided only in the Funding Statement in the online submission system.

**Funding Statement (requested update / confirmation):**

We would like the Funding Statement to read as follows:

“This research was funded by the National Natural Science Foundation of China (Grant No. 42201006) and by the Open Fund (DGERA 20221105) of the Key Laboratory of Deep-time Geography and Environment Reconstruction and Applications of the Ministry of Natural Resources, Chengdu University of Technology. We confirm that the funding agencies had no role in the study design, data collection and analysis, decision to publish, or preparation of the manuscript.” **Please help us submit it via the online.**

**Requirement 6**

If the reviewer comments include a recommendation to cite specific previously published works, please review and evaluate these publications to determine whether they are relevant and should be cited. There is no requirement to cite these works unless the editor has indicated otherwise.

**Response:**

We have evaluated all reviewer-suggested references and incorporated those that are relevant to strengthening the manuscript’s regional tectonic and geological background and interpretation. References not directly relevant were not added.

# Response to Reviewer #1

We thank you for the constructive and insightful comments. We have revised the manuscript accordingly. Major revisions include: (i) expanding the discussion of transient high-temperature heating and its implications for zircon (U–Th)/He resetting; (ii) clarifying the interpretation of the Middle Pleistocene age cluster versus isolated older ages and explaining how these age populations are identified and interpreted; (iii) adding discussion of non-tectonic controls on coal-seam combustion (coal rank, oxygen supply, aridity, ignition sources) and clarifying how tectonic vs. environmental triggers are treated; and (iv) strengthening the regional tectonic synthesis by comparing our ages to published thermochronologic constraints from nearby Bogda Shan.

**Comment 1**

The paper assumes that the combustion process completely resets the zircon (U-Th)/He system. However, CM rocks typically experience very high temperatures (up to >1000 °C) but over short durations (hours to days), leading to highly non-equilibrium diffusion conditions. The authors should provide a more quantitative discussion on whether such transient heating events can fully reset zircon He ages. Incorporating diffusion models or referencing kinetic studies would help justify the reliability of the obtained ages.

**Response:**

We agree that transient heating and non-equilibrium conditions are important when interpreting zircon (U–Th)/He ages from combustion metamorphic (CM) settings. In the revised manuscript, we expanded the discussion of reset/partial-reset behavior under short-duration, high-temperature heating by incorporating empirical/kinetic constraints and published applications of ZHe in CM rocks.

Specifically, we added text noting (i) combustion temperatures can exceed 1000 °C and may persist for long durations in natural coal-seam fires (reported to last up to hundreds of years), which increases the likelihood of substantial He loss; and (ii) empirical studies of brief, high-temperature heating (e.g., wildfire analogs) demonstrate strong He loss in apatite (>90%) but more limited He loss in zircon (~15%), highlighting that zircon can retain inherited signals under short events and thus motivating careful interpretation of single-grain outliers.

We also added the kinetic context that, under relatively short-duration high-temperature conditions, zircon fission-track annealing can be faster than He diffusion (Reiners, 2005), which is widely used as a diagnostic framework for transient reheating and partial resetting.

Finally, we now explicitly interpret our results in light of this framework: the tight Middle Pleistocene cluster is taken to reflect effective resetting for most grains, whereas isolated older grains are treated as incompletely degassed/inherited (see Response to Comment 2).

(see lines 73–89 in the revised manuscript)

**Comment 2**

The manuscript reports a Middle Pleistocene (~0.63 Ma) age and additional Late Miocene ages. It is unclear whether these reflect multiple combustion episodes, partial resetting, or sample mixing. A more detailed explanation is needed on how these age populations were identified, and what geological processes they represent. In particular, how can the Late Miocene event be reconciled with the much younger Pleistocene age - do they correspond to different tectonic stages, or to local reactivation and erosion events?

**Response:**

We agree and have clarified both (i) how the age populations are identified and (ii) what geological processes they represent.

In our dataset, three zircon grains yield a tightly clustered Middle Pleistocene population (0.56–0.70 Ma; weighted mean 0.63 ± 0.19 Ma). In contrast, two grains yield isolated older ages (8.7 ± 0.5 Ma and 87.7 ± 5.4 Ma) that are single-grain results and strongly separated from the cluster. We therefore interpret the older ages not as a combustion episode, but as inherited thermochronologic signals retained due to incomplete degassing/partial resetting during the younger combustion event (especially plausible under transient heating).

To reconcile the Late Miocene signal with the Middle Pleistocene combustion age, we explicitly frame them as recording different stages of the regional uplift–exhumation history: Late Miocene uplift and denudation is widely documented around the eastern Junggar margin (Bogda and Kelameili), and this earlier phase could be preserved in incompletely reset grains; continued uplift and erosion later culminated in Middle Pleistocene near-surface exposure of Jurassic coal seams, triggering the combustion event recorded by the clustered ~0.63 Ma ages.

We also revised the abstract to clearly state that the older ages are interpreted as inherited signals rather than a definitive separate combustion event.

(see lines 300–303、366-375 and 405-416 in the revised manuscript)

**Comment 3**

While CM rock formation is indeed associated with near-surface exposure, it may also depend on factors such as coal rank, oxygen supply, aridity, and fracture permeability. Therefore, linking combustion ages directly to tectonic uplift could oversimplify the process. The authors should discuss possible non-tectonic controls and clarify how they distinguished tectonic from environmental triggers.

**Response:**

We agree and have expanded the discussion of non-tectonic controls. In the revised manuscript we now explicitly describe external and environmental factors including potential ignition sources (e.g., lightning/wildfires), arid–semi-arid conditions favoring long-term exposure and oxidation, and the role of groundwater/overburden in suppressing combustion under humid conditions.

(see lines 340–357 in the revised manuscript)

At the same time, we clarify our logic for relating combustion ages to tectonics: uplift and denudation are treated as the necessary boundary condition that brings coal seams to shallow, oxygen-accessible conditions, while permeability/fracturing pathways (faults/joints) and climate modulate the probability and persistence of combustion. We therefore avoid claiming a one-to-one deterministic link (“age = uplift time”), and instead interpret CM ages as a near-surface exposure/oxidation threshold marker that is most reasonably coupled to uplift/erosion in this structural context.

(see lines 368–375 and 430-435 in the revised manuscript)

**Comment 4**

The regional tectonic evolution of the eastern Junggar belt should be described more comprehensively. How do the derived ages compare with other thermochronological or geomorphic constraints from the nearby Bogda Shan, Kelameili, or Karamay regions? Including a regional synthesis (e.g., with published AFT, AHe, or sedimentary record data) would enhance the geological significance of this study.

**Response:**

We agree and have strengthened the regional synthesis. In the revised manuscript, we added a more comprehensive comparison with published constraints from adjacent regions. For example, we now summarize published AFT-based uplift/denudation phases from the northern Bogda Mountains (e.g., 12–7 Ma and since ~11 Ma) and from the Kelameili Mountains (rapid uplift/denudation since ~20 Ma), and we emphasize that many basin-margin thermal history models indicate accelerated uplift since ~10 Ma.

(see lines 405–410 in the revised manuscript)

To present this clearly, we compiled these regional constraints together with our CM-rock ages in a synthesis figure (Fig. 6) to highlight where our Late Miocene inherited signal and Middle Pleistocene combustion cluster fit within the broader eastern Junggar tectonic evolution.

# Response to Reviewer #2

We sincerely thank you for your constructive and detailed comments, which helped us substantially improve the clarity, geological context, and interpretation of our dataset. We have carefully revised the manuscript accordingly. Below we provide a point-by-point response, with all changes implemented in the revised version.

**Comment 1**

Abstract: Please add more details regarding the data and results.

**Response:**

We have revised the Abstract to include more dataset- and result-specific information, including (i) the types of data collected (coal petrography/proximate–ultimate analyses; petrography and XRD mineralogy of CM rocks; zircon (U–Th)/He dating), and (ii) the key numerical result (clustered Middle Pleistocene ZHe age, 0.63 ± 0.19 Ma) and the presence/interpretation of isolated older single-grain ages.

(see lines 24–34 in the revised manuscript)

**Comment 2**

Line 53: The abbreviation is not necessary.

**Response:**

We agree and have removed the unnecessary abbreviation at its first occurrence and/or replaced it with the full term where it is not needed for readability.

**Comment 3**

Line 91: please add one or two sentence to introduce the tectonic setting or the regional geology of the Junggar basin…

**Response:**

We added a concise introduction to the regional tectonic/geomorphic setting of the Junggar Basin and its peripheral orogenic belts in the Introduction, and we strengthened the linkage to the eastern Junggar structural framework in the Geological Setting. The study area is now explicitly placed within the eastern uplift of the Junggar Basin, bounded by the Kelameili Mountains to the north and the Bogda Mountains to the south, and within the Shazhang Fault–Fold Belt structural subdivision. Relevant references (including those suggested by the reviewer) have been incorporated.

(see lines 111、117-118 and 122 in the revised manuscript)

**Comment 4**

Line 101: “it”, please specify.

**Response:**

We revised the sentence by explicitly stating the antecedent of “it”，“Shazhang Fault–Fold Belt”

(see line 117 in the revised manuscript)

**Comment 5**

Line 142: Which study area? Please specify clearly.

**Response:**

The study area is now explicitly defined as the Shazhang Fault–Fold Belt.

(see line 159 in the revised manuscript)

**Comment 6**

Line 198: Section 4.1.1; Line 206: Section 4.1.2; Line 215: Section 4.1.3.

**Response:**

Thank you for noting this. We corrected the internal cross-references and ensured they point to the correct subsections (4.1.1–4.1.3) in the revised manuscript.

(see lines 219、228 and 237 in the revised manuscript)

**Comment 7**

Section 4.3: “and another 87.7 ± 5.4 Ma; due” — how should this be interpreted? Why was this data point discarded?

**Response:**

We revised Section 4.3 and the Discussion to clearly explain the interpretation and treatment of the two isolated older single-grain ages (8.7 ± 0.5 Ma and 87.7 ± 5.4 Ma). Specifically:

Main age population: Three grains yield a tight cluster (0.56–0.70 Ma) with a weighted mean of 0.63 ± 0.19 Ma, interpreted as the timing of the combustion event that formed the CM rocks.

Isolated older ages: The 8.7 Ma and 87.7 Ma results occur as single-grain ages, strongly separated from the clustered Pleistocene population. We therefore do not include them in the weighted mean and treat them as inherited/partially reset signals, likely reflecting incomplete He loss during a short-lived heating event and/or inherited thermochronologic information.

We also clarified wording so the reader understands these data were not “discarded” as errors, but rather excluded from the mean age calculation because they are not part of the principal combustion-reset age population.

(see lines 301–303 、366-375 in the revised manuscript)

**Comment 8**

Section 5.1: Are these strata currently undergoing spontaneous combustion, or did combustion occur in the Cenozoic?… If combustion temperature exceeded the closure temperature… would that imply thermochronologic ages might not reflect uplift or exhumation?

**Response:**

We appreciate this important point and expanded Section 5.1 and the interpretation framework to avoid overstatement. Our revised text clarifies that:

The ZHe ages from CM rocks primarily date the combustion-related heating (and associated exposure to oxygen-rich near-surface conditions), rather than directly and uniquely dating a tectonic “event.” The linkage to uplift/exhumation is interpretative, based on the mechanism that coal seams typically ignite shortly after being exhumed to near-surface conditions.

(see lines 340–357 in the revised manuscript)

We explicitly discuss that combustion temperatures can be extremely high and can reset low-temperature thermochronometers; therefore, the clock records the time of thermal resetting by combustion, not necessarily the onset of uplift. We added/strengthened the explanation using the closure-temperature context already provided (apatite ~70 °C; zircon ~180–200 °C) and the transient high-temperature heating literature basis.

(see lines 73–89 in the revised manuscript)

The presence of single-grain older ages is used to support the possibility of partial resetting in some zircons during short-lived heating, which is why we interpret them as inherited/partially degassed rather than separate combustion episodes.

In addition, we revised the narrative to emphasize that our contribution is to provide a high-resolution chronological marker for near-surface exposure/combustion and then discuss how this constrains the timing of exposure associated with late Cenozoic uplift and denudation, rather than equating thermochronologic ages one-to-one with tectonic events.

(see lines 348–357 in the revised manuscript)

**Comment 9**

Section 5.1, second-to-last paragraph: …With only two sentences, it does not read as a complete paragraph.

**Response:**

We agree and have merged this short paragraph with the adjacent paragraph(s) to improve logic and readability.

(see lines 420–435 in the revised manuscript)

**Comment 10**

Figure 1: Axial traces are generally shown in black, and faults in red. Please also add representative attitudes (strike and dip) of strata.

**Response:**

We revised Figure 1 following standard geological cartographic conventions: axial traces are now shown in black and representative bedding attitudes. The Figure 1 caption was also updated accordingly.

(see line 124 in the revised manuscript)

**Comment 11**

Figure 2: Which study area? Please specify.

**Response:**

We revised the Figure 2 caption to explicitly indicate that the stratigraphic column refers to the Shazhang Fault–Fold Belt

(see line 159 in the revised manuscript)

**Comment 12**

Figure 6: More detailed and case-specific interpretation is needed… ages do not necessarily directly represent the timing of tectonic events.

**Response:**

We revised the text associated with Figure 6 and the Discussion to provide a more careful, case-specific interpretation. In particular, we now explicitly state that low-temperature thermochronology ages are not automatically equivalent to discrete tectonic events; instead, they record cooling histories that may reflect combinations of exhumation, geothermal-structure changes, and/or transient heating. We therefore use Figure 6 as regional context and avoid over-interpreting single age signals as unique tectonic episodes.

(see line 418 in the revised manuscript)

**Comment 13**

Figure 7: If these data were not collected in this study… they should be moved to the Geological Background section.

**Response:**

We agree. We moved Figure 7 to the Geological Background (Figure 1)

(see line 124 in the revised manuscript)

**Comment 14**

Section 6: A subheading (1) is not necessary…

**Response:**

We removed the unnecessary “(1)” subheading and revised the sentence to serve directly as the topic sentence of the paragraph in the Conclusions.

(see lines 437-451 in the revised manuscript)
