# Supplementary material for: Late Cenozoic exhumation in the eastern Junggar Basin: Evidence from zircon (U-Th)/He ages of combustion metamorphic rocks
Source: PLoS One. 2026 May 5;21(5):e0348116. doi: 10.1371/journal.pone.0348116 (PMC13143060; doi:10.1371/journal.pone.0348116)
Supplement: Attachment — Submitted filename: Response_to_Reviewers_auresp_2.docx [file pone.0348116.s004.docx]

**Response to Reviewers**

**Manuscript title:** Late Cenozoic exhumation in the eastern Junggar Basin: Evidence from zircon (U-Th)/He ages of combustion metamorphic rocks

**Manuscript ID:** PONE-D-25-53522R1

We sincerely thank the Academic Editor and the reviewers for their careful evaluation of our manuscript and for the constructive suggestions that helped us improve the paper. We have revised the manuscript accordingly and carefully polished the language throughout. Our detailed responses are provided below.

**Reviewer #1**

1. Although the authors appropriately discuss partial resetting and inherited signals, the zircon (U-Th)/He dataset remains relatively small. It may be helpful to add a brief statement in the Discussion acknowledging the limited number of dated grains and noting that future studies with larger datasets would further test and refine the proposed interpretation.

**Response:** We agree and have added an explicit limitation statement in Section 5.2. The revised text now notes that only five usable single-grain ages were obtained from a single sample, that the present dataset is not sufficient for robust inverse thermal-history modelling, and that future work using larger single-grain datasets together with zircon imaging and complementary thermochronometers will be necessary to refine the interpretation of the anomalously old grains.

(see lines 377-385, 415-451 in the revised manuscript)

2. The manuscript refers to long-lasting coal-fire events in some settings. Where possible, the authors may wish to clarify whether such durations are directly documented in the study area or inferred from analogs, in order to avoid potential overgeneralization.

**Response:** We agree and have revised the relevant sentence in the Introduction. In the original manuscript, the statement that coal-fire-related heating may persist for hundreds of years was based on published studies from other regions, particularly the Powder River Basin, rather than on direct chronological or observational constraints from the eastern Junggar Basin. We have therefore revised the text to make this distinction explicit. In the revised manuscript, we now clarify that prolonged coal-fire durations are documented in analogous natural coal-fire systems elsewhere, whereas in our study area the available evidence primarily constrains the timing of combustion metamorphic rock formation rather than the exact duration of individual combustion events. This revision avoids overgeneralization and better reflects the limits of the present dataset.

(see lines 73-77 in the revised manuscript)

3. Please ensure that Figs. 4 and 6 and related synthesis figures are sufficiently clear and readable at publication scale.

**Response:** We have checked the figure captions and manuscript references carefully with a simplified layout suitable for publication scale.

(see lines 282, 431 in the revised manuscript)

4. A careful proofreading is recommended to correct minor typographical errors and improve sentence flow in several places (e.g., in the Abstract and Section 5).

**Response:** We have carefully proofread the entire manuscript and revised the Abstract and Section 5 for clarity, concision, and sentence flow. Minor typographical issues, capitalization inconsistencies, and wording problems were corrected throughout.

(see lines 14-32 and 360-388 in the revised manuscript)

**Reviewer #2**

1. Fig.1: fold-thrust belt; Anticline. Please double check throughout the manuscript.

**Response:** We have double-checked the terminology throughout the manuscript and standardized capitalization and usage of structural names. In particular, occurrences such as 'Huoshaoshan anticline' and 'Xidagou syncline' were revised to 'Huoshaoshan Anticline' and 'Xidagou Syncline' where they refer to formal structural units. In the manuscript text, we have also standardized the terminology by changing 'Fault-Fold Belt' to the proper format 'Fold-Thrust Belt'.

2. The ages of 8.7 Ma and 87.7 Ma are interpreted as 'inherited zircon (U-Th)/He ages,' but no supporting evidence is provided. For example, no analyses of zircon morphology, inclusions, or U-Th distribution were conducted to exclude the effects of crystal heterogeneity. The possibility that these ages represent partially reset residual ages is not discussed, nor is it stated whether thermal history modeling (e.g., HeFTy or QTQt) was performed to test the possibility of multi-stage cooling. If possible, it is recommended to include correlations between single-grain zircon U-Th contents, eU values, and ages to evaluate whether the anomalous ages are related to radiation damage.

**Response:** We appreciate this important suggestion and have revised both the Methods and Discussion accordingly. First, we now state explicitly in Section 3.7 that the dated zircons were clear, euhedral, inclusion-free under binocular and polarized-light inspection, and that all dated grains are euhedral zircons with two terminations. Second, we now clarify that no cathodoluminescence imaging, internal U-Th zoning analyses, or inverse thermal-history modelling (e.g., HeFTy/QTQt) were performed, and we explain that the available dataset is too limited to support robust thermal-history inversion. Third, we revised the interpretation of the older grains to be more cautious: rather than treating them unequivocally as inherited ages, we now interpret them as incompletely reset or inherited pre-combustion thermochronologic components. Finally, we added an eU-age comparison and discuss its implications in the Results and Discussion.

(see lines371-385, 415-451 in the revised manuscript)

3. Zircon (U-Th)/He ages are commonly influenced by radiation damage, which typically results in a negative correlation between eU and age. However, the manuscript does not present an eU-age plot, making it difficult to assess whether the age dispersion is related to eU. If possible, the authors are encouraged to add an eU-age scatter plot and discuss whether differential He retention due to radiation damage accumulation could explain the observed age variability.

**Response:** We agree and have expanded this point in the revised manuscript. We further compared the single-grain zircon (U-Th)/He ages with the U and Th contents reported in Table 2. These comparisons show similarly non-systematic patterns: the 87.7 Ma grain does not have the highest U or Th content, the 8.7 Ma grain has the highest eU value, and the three Middle Pleistocene grains remain tightly clustered in age despite substantial variation in U, Th, and eU. We therefore interpret the age dispersion cautiously and conclude that radiation damage alone is insufficient to explain the observed spread. Instead, the two anomalously older grains are more plausibly interpreted as incompletely reset or inherited pre-combustion components. We also now state explicitly that, because only five usable grains were obtained from a single sample, these age-composition relationships should be regarded as exploratory rather than statistically robust.

(see lines 366-376 in the revised manuscript)

We again thank the reviewers for their constructive comments, which have substantially improved the manuscript.
